# DEVELOPING AN AGE-FRIENDLY ECOSYSTEM: A CASE STUDY FROM MINNESOTA’S MULTISECTOR BLUEPRINT ON AGING

**DOI:** 10.1093/geroni/igad104.2360

**Published:** 2023-12-21

**Authors:** Farah Baig, Rajean Moone, Carrie Graham

**Affiliations:** University of Minnesota, Minneapolis, Minnesota, United States; University of Minnesota, Minneapolis, Minnesota, United States; Center for Health Care Strategies, Hamilton, New Jersey, United States

## Abstract

As age demographics continue to shift, age-friendly policy initiatives at the global, national, state, and local levels are rapidly evolving. These emerging age-friendly initiatives underscore a changing perception of aging and older adulthood. Many states, communities, academic institutions, health systems, and other organizations are embracing the demographic shift and leveraging age-friendly frameworks that seek to improve later life experiences. However, there is limited scholarly work that explores contextualized age-friendly policy development. Using qualitative case study and historiography methods, this study provides an overview of various age-friendly frameworks and outlines work in the State of Minnesota where intersections of the various frameworks are coalescing in an age-friendly ecosystem. Minnesota, rather than being a ‘best practices’ exemplar, serves as a model of an evolving system, led by the Age-friendly Minnesota Council in partnership with organizations such as the Minnesota Board on Aging and the Minnesota Department of Human Services, that are developing impactful policies to address the needs of a growing heterogeneous older adult population. The nuances and realities found within the Minnesota context underscore the need to develop fluid age-friendly ecosystems that can adapt to the uniqueness and complexity found in each state, region, or community. Going forward, it will be critical for Minnesota to evaluate their programs to determine their long term impact on the entire population — including today’s older adults as well as future older Minnesotans.

